# Prehabilitation for frail patients undergoing hip and knee replacement in the UK: Joint PREP feasibility study for a randomised controlled trial

**DOI:** 10.1136/bmjopen-2024-084678

**Published:** 2024-09-17

**Authors:** Tanzeela Khalid, Yoav Ben-Shlomo, Wendy Bertram, Lucy Culliford, Emily J Henderson, Marcus Jepson, Emma Johnson, Alex Mitchell, Shea Palmer, Jonathan Thomas Evans, Michael R Whitehouse, Vikki Wylde

**Affiliations:** 1 Bristol Medical School, University of Bristol, Bristol, UK; 2 National Institute for Health and Care Research Applied Research Collaboration West at University Hospitals Bristol and Weston NHS Foundation Trust, United Kingdom, Bristol, UK; 3 National Institute for Health and Care Research Bristol Biomedical Research Centre, University Hospitals Bristol and Weston NHS Foundation Trust and University of Bristol, Bristol, UK; 4 Bristol Trials Centre, Bristol, UK; 5 School of Healthcare Sciences, Cardiff University, Cardiff, UK; 6 University of Exeter Medical School, University of Exeter, Exeter, UK

**Keywords:** frailty, hip, knee, feasibility studies

## Abstract

**Objective:**

To determine the feasibility of conducting a randomised controlled trial (RCT) to evaluate a prehabilitation programme for frail patients undergoing total hip replacement (THR) or total knee replacement (TKR).

**Design:**

Randomised feasibility study with embedded qualitative work.

**Setting:**

Three National Health Service hospitals.

**Participants:**

Adults aged ≥65 years, frail and scheduled for primary THR or TKR.

**Intervention:**

Appointment with a physiotherapist to individualise a home-based exercise programme. Participants were encouraged to do the home exercises daily for 12 weeks pre-operative and were provided with a daily protein supplement. Participants were supported by six telephone calls over the 12-week intervention period.

**Outcome measures:**

Eligibility and recruitment rates, intervention adherence, data completion rates of patient-reported outcome measures, retention rates and acceptability of the trial and intervention. Qualitative interviews were conducted with participants and non-participants and analysed using thematic analysis.

**Results:**

Between December 2022 and August 2023, 411 patients were sent a screening pack. Of the 168 patients who returned a screening questionnaire, 79 were eligible and consented to participate, and 64 were randomised. Of the 33 participants randomised to the intervention, 26 attended the intervention appointment. Eighteen participants (69%) received all six intervention follow-up telephone calls. Nineteen participants (73%) completed an intervention adherence log; 13 (68%) adhered to the exercise programme and 11 (58%) adhered to the protein supplementation. The overall retention rate was 86% (55/64 overall) at 12 weeks. The 12-week follow-up questionnaire was returned by 46 of the 55 participants (84%) who were sent a questionnaire. Interviews with 19 patients found that the trial processes and intervention were generally acceptable, but areas of potential improvements were identified.

**Conclusions:**

This study demonstrated that a larger study is possible and has identified improvements to optimise the design of an RCT.

**Trial registration number:**

ISRCTN11121506.

**STRENGTHS AND LIMITATIONS OF THIS STUDY**

- Patient representatives were involved throughout the study, from design to dissemination.
- The study was delivered across three National Health Service hospital sites, therefore demonstrating the feasibility of a future multicentre randomised controlled trial.
- Assessment of intervention adherence was limited as not all participants randomised to the intervention group returned a complete adherence log.
- Study sites served populations with limited ethnic diversity; therefore, cautious interpretation of study generalisability is warranted.

## Background

 Osteoarthritis is a leading cause of chronic pain and disability globally.^1^ Joint replacement aims to provide relief from chronic pain and improve functional ability.^2 3^ Total hip replacement (THR) and total knee replacement (TKR) are two of the most common elective surgical procedures, with over >200 000 performed annually in the National Health Service (NHS).^4 5^ The average age of people having a joint replacement in the UK is 70 years^5^ and approximately 20%–25% have moderate-to-severe frailty and another 40%–45% have mild frailty.^6^ Frailty is an age-related condition associated with a deterioration in the physiological capacity of multiple organ systems that causes an increased susceptibility to physiological stressors, such as illness and surgery. A future increase in the prevalence and severity of frailty among patients having joint replacement in the NHS is likely due to an ageing population and the deterioration in health associated with a long surgical waiting time.^7^

Frailty is associated with increases in mortality rate, risk of admission to intensive care, length of hospital stay, risk of being discharged to institutional care and readmissions to hospital; and with poorer patient-reported outcomes after joint replacement.^68^,^11^ Nonetheless, patients with frailty often experience improvements in pain and function after joint replacement.^6^ Importantly, frailty is potentially modifiable, with physical inactivity and inadequate nutrition being important contributors to frailty.^12^ The mechanistic pathway for a combined approach of exercise and protein in frailty is that exercise sensitises muscles to dietary protein, resulting in more of the available amino acids being synthesised into skeletal muscle protein.^13^ The existing literature provides evidence for proof of concept that exercise combined with protein supplementation can improve health in people with frailty. Systematic reviews have found that a combination of protein and exercise are associated with improvements in lean mass, muscle strength and function in frail people.^14 15^ A recent randomised controlled trial (RCT) of an exercise and dietary protein intervention for frail adults found the intervention reduced frailty and improved general health.^16^ A series of *Lancet* articles on the management of frailty highlights that all recent consensus-based guidelines have included physical activity and adequate protein intake as first-line therapies for the management of frailty.^17 18^ In a surgical context, systematic reviews and meta-analyses have found that prehabilitation improves function and reduces length of hospital stay and severe post-operative complications for frail patients undergoing surgery for cancer and elective abdominal surgery.^19^,^21^ However, the effectiveness of prehabilitation for frail patients undergoing THR or TKR has not been evaluated.

Robust evaluation of prehabilitation programmes comprising exercise and protein supplementation is needed to inform orthopaedic care provision for frail patients undergoing joint replacement. Prior to conducting an RCT to evaluate a complex intervention, a feasibility study can address key uncertainties regarding whether an RCT is possible and explore how to optimise trial design and delivery. Previous RCTs of interventions with an exercise component have highlighted that intervention adherence can be an issue.^22^ The aim of the study was to determine the feasibility of conducting an RCT to evaluate the clinical and cost-effectiveness of a pre-operative 12-week prehabilitation intervention comprising a tailored home exercise programme and daily protein supplementation for frail patients on the waiting list for a THR or TKR. Specific objectives were to determine eligibility and recruitment rates, intervention adherence, data completion rates, retention rates and acceptability of the trial and intervention.

## Methods

### Design

Joint PREP (Joint PRehabilitation with Exercise and Protein) is a feasibility study with embedded qualitative research for a multicentre, parallel, two-arm, pragmatic RCT with 1:1 allocation ratio. The study was conducted at three NHS hospitals based in Bristol, Cardiff and Exeter. A Consolidated Standards of Reporting Trials checklist for reporting feasibility studies^23^ is provided in the online supplemental materials.

### Study registration

The study was registered on the International Standard Randomised Controlled Trial Number registry (ISRCTN11121506) on 29 September 2022. The protocol has been published.^24^

### Patient and public involvement

This study was conducted in collaboration with a patient and public involvement group, called the Patient Experience Partnership in Research group. This is an established and experienced forum of patients who have had, or are having, treatment for musculoskeletal health conditions, including joint replacement. Patient representatives have worked with the research team on study design, delivery, interpretation and dissemination. This has included testing protein supplements and providing feedback on the exercise programme and documents; preparing patient-facing study documents including the screening questionnaire, patient information leaflet and intervention adherence log; developing the interview topic guide; aiding interpretation of how the findings could improve the design of a future RCT; deciding on the outcomes for the future RCT and helping with drafting the plain language summary of findings for participants.

### Patient recruitment

Inclusion criteria were patients scheduled for primary THR/TKR, ≥12 weeks until intended date of operation, ≥65 years of age and frail according to the self-reported Groningen Frailty Indicator (GFI; score of ≥4).^25^ The GFI is a 15-item multidimensional screening tool for frailty, with questions covering daily activities, health problems and psychosocial functioning. Exclusion criteria were contraindications to study treatment or participation in another study that could affect outcomes or where participation would be burdensome to the patient; this was considered on a case-by-case basis.

Patients on the waiting list for a primary THR/TKR who were aged ≥65 years and had ≥12 weeks until intended date of operation were identified from hospital records by the clinical care team and sent a postal screening pack consisting of the study participant information leaflet, screening questionnaire and consent form. The screening questionnaire included the GFI, the self-report Clinical Frailty Scale^26^ and questions about any health conditions which may preclude exercise or taking protein supplements and participation in other studies. Interested patients completed and returned the screening questionnaire and consent form. Eligible consenting patients were telephoned by the local research team to confirm that they fully understood what participation involved and answer any questions.

### Randomisation

Participants were randomised by the local research team using a computer-based tool (Research Electronic Data Capture (REDCap)), stratified by hospital and operation (THR/TKR). Use of REDCap ensured that the allocation sequence was concealed from all study personnel and that treatment allocation was not revealed until eligibility was confirmed and the patient was randomised. Participants were informed of their treatment allocation by letter, followed by a telephone call to participants randomised to the intervention group to discuss intervention arrangements. Blinding was not possible due to the nature of the intervention.

### Usual care

All participants received usual care which comprised clinical review by a surgeon before and after surgery. Other aspects of usual care varied between NHS Trusts, but could involve education classes, physiotherapy and occupational therapy.

### Intervention

Participants randomised to the intervention group were invited to undertake 12 weeks of a prehabilitation programme, comprising pre-operative exercise and protein supplementation. The intervention was designed as a personalised and home-based programme with regular contact and support to maximise adherence.^27^ A duration of 12 weeks was informed by previous research as a minimum duration of exercise needed to have benefits relevant to frailty.^28^

#### Exercise

Participants had a single 1:1 appointment with a trained physiotherapist at the hospital to individualise a 12-week home-based exercise programme. In the protocol it was planned that the appointment would be offered in-person or via a secure video platform, but all participants opted for in-person appointment. A risk assessment was conducted to inform adaptation of exercises and progression schedule to ensure they were tailored to individual physical capabilities and to minimise risk. The exercises and resources were based on the NEMEX-TJR programme (https://nemex.trekeducation.org) and adapted for home use with permission from the Translating Research Evidence and Knowledge programme at La Trobe University, Australia.^29^ This programme is a neuromuscular training method for patients with osteoarthritis who are undergoing joint replacement.^30^ The exercises included a warmup, pelvic lifts, sit-ups, lunges, sideway lunges, knee flexion and extension, hip abduction and adduction, chair stands, step ups and a cool down. The physiotherapist discussed each exercise with the participant and the participant was encouraged to decide which level of each exercise was most appropriate for them. They then performed them with the physiotherapist who checked safety and technique. Participants were encouraged to aim for 10–15 repetitions of each exercise and 2–3 sets. Participants were provided with two booklets to assist them with completing the exercises. One booklet contained illustrations and instructions specific to the exercises; and the other booklet contained more general information such as the benefits of exercise, goal setting, pacing and dealing with setbacks. All participants were issued with TheraBand at an appropriate resistance level for their capability (special heavy, extra heavy, heavy or medium) and where appropriate, an exercise step and/or gym ball.

#### Protein

Participants were asked to consume 20 g of additional protein, in the form of one jelly pot that was low in carbohydrate and 90 calories, each day for 12 weeks (ProSource jelly; Nutrinovo). Participants who did not eat gelatine or could not tolerate the jelly were offered protein powder (Pulsin) to make protein shakes. Participants were asked to consume the protein within 3 hours after exercise as muscle protein synthesis peaks during this time.^31^ They were also advised to consume the protein between meals to minimise any effect on appetite.

#### Telephone follow-up calls

Participants were telephoned by a physiotherapist at 1, 2, 4, 6, 8 and 10 weeks to check they were managing with the exercises and protein supplements, discuss progression of exercises and address any concerns. If participants reported in a telephone follow-up call that they could confidently perform the exercises at one level, they were encouraged to consider progression to the next level. If participants reported that they were struggling with any of the exercises, they were encouraged to regress to an easier level.

#### Intervention training

The intervention was delivered by physiotherapists working clinically with orthopaedic patients. Physiotherapists (two per hospital site) attended a half-day training session with a registered dietitian and physiotherapist and were provided with an intervention manual.

### Questionnaires

Postal questionnaires were completed at baseline (before randomisation) and 12 weeks after randomisation. Questionnaires were chosen to assess the health domains that could potentially be influenced by the intervention. Non-responders were followed-up with a reminder questionnaire and then a telephone call.

Questionnaires included:

- Joint pain and function: Western Ontario and McMaster Universities Osteoarthritis Index.^32^
- General health: EuroQol-5 Dimension 5-level.^33^
- Capability: ICEpop Capability Measure for Older People.^34^
- Frailty: GFI.^25^
- Physical activity level: Global Physical Activity Questionnaire.^35^
- Exercise self-efficacy and beliefs: exercise self-efficacy and beliefs questionnaire.^36^
- Dietary protein adequacy: protein screener 55+.^37^
- Body mass index: height and weight.

In the 12-week questionnaire, the usual care group was asked about changes to usual diet or exercise to assess potential contamination, while the intervention group was asked questions about intervention acceptability. In the protocol, it was planned that the Clinical Frailty Scale would be completed by a healthcare professional when the patient attended the hospital for their routine pre-operative assessment appointment (to allow comparison of the self-reported and clinician-assessed versions of the Clinical Frailty Scale), however these data were not collected due to limited site capacity and some patients not having a pre-operative assessment appointment in the timeframe of the study.

### Data collection from medical records

Data were extracted from participants’ medical records on comorbidities, indication for surgery, surgery details, length of hospital stay, discharge destination (own home/sheltered housing, residential care, nursing care, rehabilitation, acute hospital, community hospital), whether the patient was mobilised on the day of surgery/day following surgery and complications up to 30 days post-operatively.

### Safety

Data on adverse events were collected and all serious adverse events during the intervention period were reviewed by the health organisation responsible for the research.

### Withdrawal

Participants who withdrew were invited to provide their reasons if they wished to do so.

### Qualitative study

Patients approached for the feasibility study were invited to take part in a semi-structured interview with a qualitative researcher. Interviews followed a topic guide, which covered experiences of randomisation, intervention acceptability, experience of participation and data collection methods and any barriers or enabling factors that participants experienced in adhering to the intervention. For patients who declined participation/withdrew, questions focused on reasons for declining or withdrawing. The interview topic guides are provided in the online supplemental materials.

### Feasibility outcomes

Eligibility and recruitment were assessed by collecting data on the number of eligible, approached and consented patients, alongside information on reasons for non-eligibility and non-participation. Intervention delivery was assessed through the number of appointments and telephone calls conducted. To assess intervention adherence, participants were provided with a log to keep a daily record of whether they consumed their protein supplement and completed their exercises. Participants were considered to have adhered to the intervention if they consumed the protein supplement on ≥4 days per week for at least 10 weeks and completed the exercises ≥3 days per week for at least 10 weeks (or 80% of intervention duration if the time available was shorter than 12 weeks because surgery occurred earlier than expected). This definition of adherence was chosen to reflect current clinical opinion for targeting pre-operative frailty.^38^ Acceptability of the trial and intervention was evaluated through qualitative interviews, study questionnaires, retention rates and reasons for withdrawal. Completion rates for questionnaires were calculated.

### Sample size

To meet our target of 60 participants (deemed appropriate for feasibility studies^39^) with an estimated recruitment rate of 30%, we estimated we would need to identify 200 eligible people. If we assumed 40% of those screened would be prefrail/frail and potentially eligible, we expect to actively screen 500 patients for frailty. As this is a feasibility study, we based our sample size on recruitment rate. If we identified 200 eligible patients, we could estimate a recruitment rate of 30% (ie, 60 participants) to within a 95% CI of ±6.35%.^40^

### Data management

Pseudoanonymised study data were stored in the REDCap secure online data capture system. Participants’ personal data were stored securely and were only accessible to trial staff and authorised personnel.

### Progression criteria

Prespecified progression criteria were presented in the study protocol^24^ and were pragmatically derived by the research team to demonstrate that recruitment, adherence and retention would be sufficient to deliver a future RCT^41^:

- Recruitment: >23% (lower limit of the 95% CI for a 30% recruitment rate based on 200 screened).
- Adherence: >37% (lower limit of the 95% CI for 50% adherence rate based on 60 participants).
- Retention: >70% (lower limit of the 95% CI for a 80% retention rate based on 60 participants).

If all criteria were not met, an RCT would not be considered feasible. If one or two criteria were not met, we planned to review how processes could be modified to address the shortfall.

### Analysis

Data on recruitment, adherence and retention were reported using frequencies and percentages. Participant characteristics and outcome data were summarised using means and 95% CIs, medians and IQRs or frequencies and proportions as appropriate. Audio-recordings of interviews were transcribed and anonymised. Data were analysed using thematic analysis, guided by the constant comparison method used in the Qualitative Research Integrated within Trials (QuinteT) Recruitment Intervention.^42^ A coding index, based on the interview topic guide, was used to sort the data into themes. An inductive approach was used, allowing emergent themes to alter the coding as the analysis progressed. Although coding was completed by a single researcher (EJH), to enhance the credibility of findings, a second author (MJ) reviewed all codes and met regularly with EJH to discuss, refine and agree on the development of key themes. Qualitative data analysis was assisted by NVivo software.

## Results

### Eligibility and recruitment

**Figure 1:**
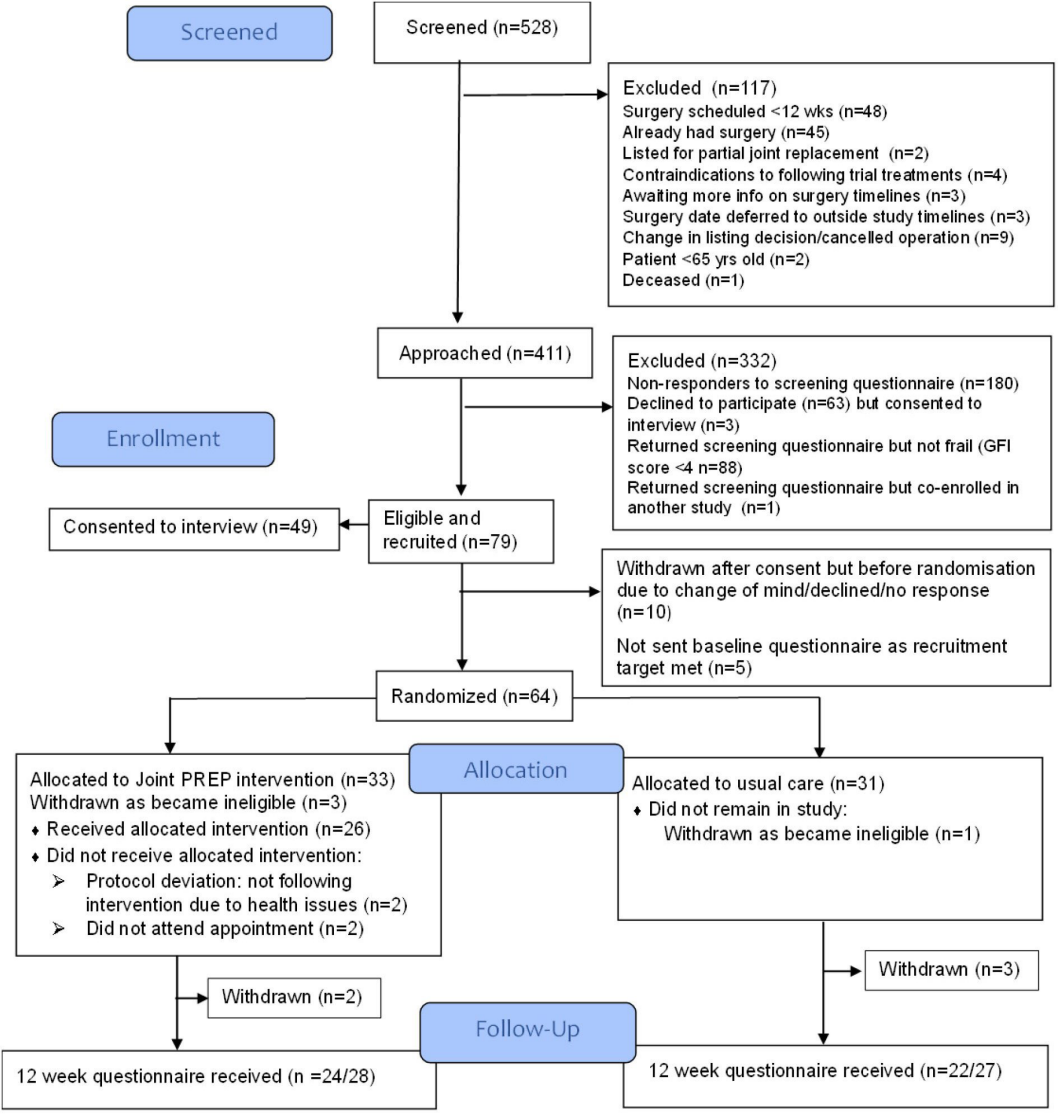
Consolidated Standards of Reporting Trials diagram. GFI, Groningen Frailty Indicator; Joint PREP, Joint PRehabilitation with Exercise and Protein.

A CONSORT diagram is provided in figure 1. Between December 2022 and August 2023, 411 patients from 3 hospitals who were ≥65 years of age, on the waiting list for a primary THR/TKR with an expected wait of >12 weeks until surgery were sent a screening pack. Of these, 168 (41%) returned a screening questionnaire and consent form. The number of patients that met the criteria for frailty (GFI score ≥4) and had no medical contraindications to the intervention was 79 (47% of returned screening questionnaires). Of these, 79 (100%) provided consent (the consent form was included in the screening questionnaire) and 64 (81%) were randomised. Reasons for ineligibility are provided in figure 1 and reasons for declining participation are provided in table 1.

**Table 1:** Reasons given for declining participation (n=63)

Reason	Number (%)
No reason given	17 (27%)
No time	17 (27%)
Too much pain	6 (10%)
Due to intervention (exercise)	6 (10%)
Due to intervention (protein)	4 (6%)
Family/Other commitments	4 (6%)
Do not feel they need support (good self-care)	3 (5%)
General health issues	3 (5%)
Do not do surveys	1 (2%)
Language barrier	1 (2%)
Want intervention will increase protein intake on own	1 (2%)

The recruitment rate was 47% (79/168), calculated as the number of eligible and consenting patients from those that returned the screening questionnaire, and the randomisation rate was 38% (64/168). The average recruitment rate in this feasibility study was 4.8 patients/site/month and the average randomisation rate was 3.9 patients/site/month.

Baseline characteristics of approached and randomised patients are provided in table 2. Randomised participants were broadly representative of all patients sent a screening pack.

**Table 2:** Baseline characteristics of participants and all approached patients

	All approached patients (n=411)	All randomised participants(n=64)	Intervention group(n=33)	Usual care group(n=31)
Mean age in years (range)	75 (65–93)	75 (65–87)	75 (65–85)	75 (65–87)
Female (%)	266 (65%)	46 (72%)	25 (76%)	21 (68%)
Ethnicity (%)				
Asian	4 (1%)	1 (2%)	0 (0%)	1 (3%)
Black	2 (0.5%)	1 (2%)	0 (0%)	1 (3%)
Mixed	1 (0.2%)	0 (0%)	0 (0%)	0 (0%)
White	277 (67%)	56 (87%)	29 (88%)	27 (87%)
Unknown	127 (31%)	6 (9%)	4 (12%)	2 (7%)
Socioeconomic deprivation (%)				
First IMD quintile—most deprived	60 (15%)	7 (11%)	2 (6%)	5 (16%)
Second IMD quintile	55 (13%)	9 (14%)	5 (15%)	4 (13%)
Third IMD quintile	77 (19%)	9 (14%)	4 (12%)	5 (16%)
Fourth IMD quintile	85 (21%)	10 (16%)	5 (15%)	5 (16%)
Fifth IMD quintile—least deprived	131 (32%)	29 (45%)	17 (52%)	12 (39%)
Unknown IMD score	3 (1%)	0 (0%)	0 (0%)	0 (0%)
Joint replacement surgery (%)				
Total knee replacement	174 (42%)	26 (41%)	13 (39%)	13 (42%)
Total hip replacement	237 (58%)	38 (59%)	20 (61%)	18 (58%)
Groningen Frailty Indicator	–			
4—moderate frailty		14 (22%)	9 (27%)	5 (16%)
5		16 (25%)	5 (15%)	11 (36%)
6		16 (25%)	9 (27%)	7 (23%)
7		6 (9%)	5 (15%)	1 (3%)
8		9 (14%)	4 (12%)	5 (16%)
9		1 (2%)	0 (0%)	1 (3%)
10—severe frailty		2 (3%)	1 (3%)	1 (3%)
Clinical Frailty Scale (self-reported)				
Missing	–	1 (2%)	0 (0%)	1 (3%)
Very fit		2 (3%)	1 (3%)	1 (3%)
Well		1 (2%)	1 (3%)	0 (0%)
Managing well		13 (20%)	6 (18%)	7 (23%)
Vulnerable		29 (45%)	19 (58%)	10 (32%)
Mildly frail		10 (16%)	4 (12%)	6 (19%)
Moderately frail		6 (9%)	2 (6%)	4 (13%)
Severely frail		2 (3%)	0 (0%)	2 (7%)
BMI (self-reported)	–			
Underweight (BMI <18.5)		0 (0%)	0 (0%)	0 (0%)
Healthy weight (BMI 18.5–25)		8 (13%)	4 (12%)	4 (13%)
Overweight (BMI 25–30)		20 (31%)	13 (39%)	7 (23%)
Obese (BMI >30)		24 (38%)	11 (33%)	13 (42%)
Missing		12 (19%)	5 (15%)	7 (23%)
WOMAC score*	–			
Total		39.9 (35.5–44.4)	42.3 (35.6–48.9)	37.1 (31.2–43.1)
Pain		40.7 (36.2–45.2)	42.3 (35.4–49.1)	39.0 (33.0–45.0)
Function		39.9 (35.2–44.7)	42.5 (35.4–49.7)	36.8 (30.6–43.1)
Stiffness		36.4 (31.6–41.3)	39.9 (32.4–47.4)	32.6 (26.5–38.7)
EQ-5D-3L^†^	–	0.25 (0.17–0.33)	0.29 (0.17–0.41)	0.21 (0.10–0.31)
EQ-5D VAS^‡^		48.2 (43.7–52.7)	48.7 (42.8–54.7)	47.6 (40.4–54.9)
ICECAP-O tariffs^§^	–			
Mean (95% CI)		0.67 (0.62–0.72)	0.67 (0.60–0.75)	0.66 (0.60–0.73)
Median		0.67	0.70	0.65
IQR		(0.55–0.81)	(0.53–0.83)	(0.60–0.9)
Global Physical Activity^¶^	–			
Missing physical activity (n, %)		7 (11%)	3 (9%)	4 (13%)
No physical activity (n, %)		31 (48%)	18 (55%)	13 (42%)
Reported doing some physical activity either at work, travel, recreational (n, %)Total METs per week		26 (41%)	12 (36%)	14 (45%)
Total who met activity guidelines of >600 METs per week		1650(480–4800)19 (30%)	1270(540–3760)9 (27%)	2400(240–4800)10 (32%)
Sedentary time (min per week)		2835(1680–3780)	3360(2520–4200)	2520(1260–3360)
Self-efficacy for exercise score^**^	–	11 (10–13)	10.5 (10-13)	12 (9–13)
Missing (n, %)		8 (13%)	3 (9%)	5 (16%)
Exercise beliefs total score (barriers, benefits and impact on arthritis)		59 (55–63)	58 (54–62)	60 (55–64)
Missing (n, %)		7 (11%)	2 (6%)	5 (16%)
Protein screener 55+^††^	–			
Missing (n, %)^‡‡^		35 (55%)	15 (46%)	20 (65%)
Predicted probability protein intake		0.40	0.60	0.20
<1.0 g/kg adj BW/d		(0.15–0.73)	(0.18–0.76)	(0.06–0.54)

* All WOMAC scores were normalizednormalised on a 0–100 scale (best to worst); values are reported as mean and 95% confidence intervalCIs.

† EQ-5D-3L: 0 is a health state equivalent to death and 1 is perfect health.

‡ EQ VAS scores are from 0 to 100 (worst to best imaginable health state); reported as mean and 95% confidence intervalCIs.

§ ICECAP-O tariffs of 1.00=full capability and 0=no capability, reported as mean (with 95% confidence intervalCIs) and median (IQR).

¶ GPAQ Total METs (Metabolic Equivalents) per week and sedentary time values are expressed as median (IQR).

** Self-efficacy for exercise and exercise belief scores are expressed as median (IQR). Scores for self-efficacy for exercise range from 5 to 20 (lowest to highest self-efficacy). Total scores for exercise beliefs range from 16 to 80, with higher scores representing more positive beliefs about exercise.

†† Scores for predicted probability of protein intake <1.0 g/kg adjusted by body weight per dayBW/d are expressed as median (IQR) and range from 0 to 1 with a higher value indicating a higher probability on a protein intake <1.0 g/kg adjusted BW/d.

‡‡ An administrative error in the response options of one of the Protein screenerProtein 55+ questions resulted in it not being possible to calculate a score for 23 participants.

adjadjustedBMIbody mass indexBW/dbody weight per dayGPAQGlobal Physical Activity QuestionnaireICECAP-OICEpop Capability Measure for Older PeopleIMDIndex of Multiple DeprivationMETsmetabolic equivalentsVASvisual analogue scaleWOMACWestern Ontario and McMaster Universities Osteoarthritis Index

### Intervention delivery

Thirty-three participants were randomised to the intervention group. Of these, three were withdrawn before being invited to an intervention appointment due to becoming ineligible because of a change in surgery date or surgery was cancelled. Of the 30 participants invited to an intervention appointment, 26 (87%) attended. Reasons for non-attendance included ongoing health issues (n=2), time constraints (n=1) and being uncontactable by telephone (n=1). Of the 26 participants who attended an appointment, 18 had all 6 telephone follow-up calls, 6 participants received 4–5 telephone calls and 3 participants received 2–3 telephone calls. The most common reason for not having all six follow-up telephone calls was due to participants having their surgery earlier than anticipated. Details on adverse events are provided in the online supplemental materials. No serious adverse events possibly related to the intervention were reported. Non-serious adverse events possibly related to the intervention were tiredness/exhaustion after exercise, joint pain, shaking, breathlessness, diarrhoea, nausea, bloating and exacerbation of pre-existing faecal incontinence.

### Intervention adherence

Adherence logs were completed by 19 participants. Of these 19 participants, 10 (53%) participants adhered to both the exercise and protein component. Adherence was slightly higher for the exercise component (13 participants; 68%) than the protein supplement component (11 participants; 58%). Reasons for non-adherence were being unwell, having minor surgery, recovering from a fall and family issues. Feedback on the intervention collected in the 12-week questionnaire is provided in table 3.

**Table 3:** Study questionnaire feedback on intervention (n=24)

	Number	%
How did you find the exercises?		
Missing	1	4
Too easy	0	0
About right	19	79
Too difficult	4	17
Were you able to do the exercises each day?		
Missing	1	4
I was able to do the exercises each day without any problems	10	42
I found it a bit difficult to do the exercises each day	8	33
I found it very difficult to do the exercises each day	5	21
How did you feel about the jelly pots/protein shakes?		
Missing	2	8
I liked the protein food/drink	10	42
I nether liked nor disliked the protein food/drink	10	42
I disliked the protein food/drink	2	8
Were you able to add the protein food or drink to your diet each day?		
Missing	3	13
I was able to add it to my diet each day without any problems	15	63
I found it a bit difficult to add to my diet each day	1	4
I found it very difficult to add to my diet each day	5	21

### Potential for contamination

Of the 22 usual care participants who returned a follow-up questionnaire, 55 participants reported increasing their intake of protein-rich foods and 6 participants reported an increase in exercise, included attending exercise classes, walking and cycling.

### Acceptability of the trial and intervention

Detailed qualitative findings will be reported separately, here we provide a summary of the findings related to the acceptability of trial processes and the intervention.

Interviews were conducted with 17 feasibility study participants. Demographics of interview participants are provided in the online supplemental materials. Most participants had been on a waiting list for joint replacement for a long time, often many years, hence their views were shaped by this experience. Patients who consented to be randomised were motivated by a willingness to help the study, and influenced by a perception that they would gain personal benefit by taking part. They reported that receiving information about the study reassured them that they were still on the waiting list, which may have been a motivating factor for participation. While participants in both groups were happy to have been allocated to treatment by randomisation, several in the usual care group believed that they had not been ‘selected’ for the study on the basis of external factors.

Participants in the intervention group highly valued the initial face-to-face appointment with the physiotherapist to assess their capabilities and explain the exercises, they also appreciated this being undertaken on a one-to-one basis rather than in a group setting. Follow-up telephone calls from the physiotherapist were also well received. Most respondents reported that the exercises were challenging to execute and that they had struggled to do as many of the exercises as they had been asked to. Some also reported that they did not feel the exercises got easier over time, hence were unable to progress to the recommended number of repetitions. Similarly, some participants were negative about their experience with the provided exercise equipment, reporting that these were too challenging for them to use, thus demotivating them and making them less likely to adhere to the exercise programme.

Most participants in the intervention group reported that they had been willing to consume the protein supplement. The majority were accepting of either the initial flavour they received or trialled a few flavours/the alternative protein option, before settling on one they preferred. However, there were mixed views about the experience of taking them, with some participants describing the taste as unpleasant. Several had mitigated for this by adding other food (eg, ice cream) to the jelly. Some reported they had not taken the protein supplement within 3 hours of exercising, instead taking the supplement with their main meal of the day.

Interviews were conducted with two patients who declined participation in the feasibility study. The decliners expressed concerns about potential side effects of the supplement, not wishing to take on the extra food or drink, or because of a general feeling that they had too much going on in their lives.

### Data completion rates and outcome measures

A baseline questionnaire was completed by 61/64 (95%) participants. Of the 55 participants who were still in the study at 12 weeks and were sent a follow-up questionnaire, 46 (84%) returned a completed questionnaire. Outcome measures from the 12-week questionnaire are summarised in table 4. No formal statistical comparisons have been made between study arms as this was a feasibility study.

**Table 4:** Outcome measures in the 12-week questionnaire for the 46 participants who returned a questionnaire

	All participants (n=46)	Intervention group (n=24)	Usual care group (n=22)
Groningen Frailty Indicator			
1–3 (able, not clinically frail)	5 (11%)	3 (13%)	2 (8%)
4—moderate frailty	6 (13%)	3 (13%)	3 (13%)
5	14 (30%)	8 (33%)	6 (25%)
6	7 (15%)	4 (17%)	3 (13%)
7	5 (11%)	1 (4%)	4 (17%)
8	6 (13%)	4 (17%)	2 (8%)
9–11—severe frailty	2 (4%)	1 (4%)	1 (4%)
Missing	1 (2%)	0 (0%)	1 (4%)
BMI (self-reported)			
Underweight (BMI <18.5)	0 (0%)	0 (0%)	0 (0%)
Healthy weight (BMI 18.5–25)	4 (9%)	2 (8%)	2 (9%)
Overweight (BMI 25–30)	18 (39%)	11 (46%)	7 (32%)
Obese (BMI >30)	21 (46%)	9 (38%)	12 (55%)
Missing	3 (6%)	2 (8%)	1 (4%)
WOMAC score*			
Total	37.8 (32.4–46.9)	39.6 (33.3–49.0)	36.9 (32.4–42.7)
Pain	40.0 (30.0–50.0)	44.4 (35.0–52.5)	40.0 (25.0–50.0)
Function	37.9 (31.3–45.6)	41.2 (32.4–48.5)	36.8 (31.3–42.6)
Stiffness	37.5 (25.0–50.0)	37.5 (25.0–50.0)	31.3 (25.0–37.5)
EQ-5D-3L†	0.27 (0.19–0.35)	0.31 (0.19–0.43)	0.22 (0.11–0.34)
EQ-5D VAS‡	53.8 (49.1–58.4)	51.0 (44.8–57.3)	56.9 (49.7–64.1)
ICECAP-O tariffs§			
Mean (95% CI)	0.72 (0.67–0.77)	0.73 (0.67–0.79)	0.70 (0.61–0.79)
Median (IQR)	0.76 (0.63–0.83)	0.79 (0.63–0.83)	0.73 (0.63–0.85)
Global Physical Activity¶			
Missing physical activity (n, %)	5 (11%)	2 (8%)	3 (14%)
No physical activity (n, %)	24 (52%)	14 (59%)	10 (45%)
Reported doing some physical activity either at work, travel, recreational (n, %)	17 (37%)	8 (33%)	9 (41%)
Total METs per week**	900 (240–3080)	1050 (180–2560)	720 (480–3080)
Total who meet activity guidelines of >600 METs per week	12 (19%)	5 (21%)	7 (32%)
Sedentary time (min per week)	3360 (2240–4074)	3360 (2520–4200)	3360 (2100–3780)
Self-efficacy for exercise score**	11 (8–14)	10.5 (7-14)	12 (9–15)
Missing (n, %)	7 (15%)	2 (8%)	5 (23%)
Protein screener 55+††			
Missing (n, %)§§	7 (15%)	5 (21%)	2 (9%)
Predicted probability protein intake <1.0 g/kg adj BW/d	0.46 (0.06–0.84)	0.61 (0.12–0.84)	0.10 (0.05–0.69)

* All WOMAC scores were normalizednormalised on a 0–100 scale, where 100=worse pain, stiffness, and functional limitations and 0=better health state; values are expressed as median (IQR).

† EQ-5D-3L is the health state index score where 0 is a health state equivalent to death and 1 is perfect health.

‡ EQ VAS scores are expressed as mean and 95% confidence intervalCIs, where 0 represents the worst imaginable health state and 100 the best imaginable health state.

§ ICECAP-O tariffs of 1.00=full capability and 0=no capability, reported as mean (with 95% confidence intervalCIs) and median (IQR).

¶ GPAQ Total METs (Metabolic Equivalents) per week and sedentary time values are expressed as median (IQR).

** Self-efficacy for exercise scores are expressed as median (IQR) and range from 5 to 20 with higher scores representing higher self-efficacy.

†† Scores for predicted probability of protein intake <1.0 g/kg adjusted by body weight per dayBW/d are expressed as median (IQR) and range from 0 to 1 with a higher value indicating a higher probability on a protein intake<1.0 g/kg adjusted BW/d.

adjadjustedBMIbody mass indexBW/dbody weight per dayGPAQGlobal Physical Activity QuestionnaireICECAP-OICEpop Capability Measure for Older PeopleMETsmetabolic equivalentsVASvisual analogue scaleWOMACWestern Ontario and McMaster Universities Osteoarthritis Index

Data about operations and hospital stay were extracted from medical records for 16 participants. This low number was due to limited site staff capacity to extract these data and some participants not having received surgery by the end of the study. Given the limited dataset, we have not reported the findings in this article.

### Retention

The retention rate was 86% (55/64) at 12 weeks; nine participants were withdrawn after randomisation due to being ineligible (surgery cancelled or surgery date scheduled for early than expected and therefore insufficient time to deliver the intervention) or patient choice. Reasons for withdrawals are provided in the online supplemental materials.

## Discussion

Through conducting a feasibility study with 64 randomised participants, we have demonstrated that an RCT of a prehabilitation programme for frail patients waiting for THR or TKR is feasible and acceptable to patients. All the progression criteria for demonstrating the feasibility of an RCT were met, with a recruitment rate of 47%, an intervention adherence rate of 53% and a retention rate of 86%. The study has identified several important adjustments that are needed to optimise the design of a future RCT. In particular, the exercises were too challenging for many participants, highlighting the need to tailor the exercise programme to the individual ability of patients with frailty, such as providing easier exercises options, incorporating regressions and graded approaches to the exercises and providing alternatives to the use of equipment as needed. In addition, the qualitative findings suggest that adherence to the protein supplements could potentially be increased through exploring additional suggestions on how to make the protein supplements more palatable.

A key strength of this study is that it addresses the priorities of patients. The James Lind Alliance Priority Setting partnership (JLA PSP) top 10 research priorities for joint replacement includes a question on which health service factors can be modified to influence post-operative outcomes and the JLA PSP top 10 research priorities for frailty includes evaluating the impact of exercise and physical activity on frailty.^43^ While JLA PSPs include representation from patients, it has been acknowledged that these formal initiatives often impose barriers to involvement of people from marginalised communities.^44^ We have worked with people of South Asian, Black, African or Caribbean heritage in the UK to identify musculoskeletal research priorities and one of the priorities identified was research into how joint symptoms can be effectively managed while waiting for joint replacement.^45^

The limitations of the study should be acknowledged when interpretating the findings. Our sample size was small, however we followed published guidance on calculating sample sizes for feasibility studies.^39^ As with many prehabilitation interventions, blinding of the intervention was not possible, which could lead to an overestimate of the treatment effect in a future RCT.^46^ Information on the usual pre-operative care provided to participants in the study was not collected and therefore we are unable to provide a detailed description on the prehabilitation that participants received. Our assessment of intervention adherence was limited as not all participants randomised to the intervention group returned their self-completed adherence log. We opted to use self-complete logs as this is one of the most commonly used methods to collect exercise adherence data,^47^ however it is acknowledged that collecting robust data on adherence to home-based interventions is challenging as self-reported adherence data are open to recall bias and overestimation of adherence.^22^ Although our progress criteria for intervention adherence was met, methods to optimise this in a future RCT are warranted. Our choice of patient-reported outcome measures to assess function reflects that the lack of patient-centred outcomes is a limitation of previous trials evaluating interventions for frailty.^17^ While we acknowledge that more objective assessments of function are valuable outcomes, our patient and public involvement group recommended that self-report function would be the most meaningful outcome to patients in a future RCT.

Reflection on the diversity of the study population is warranted. We demonstrated an ability to recruit people from ethnic minority groups, however the study sites served populations with limited ethnic diversity^48^ and one site did not routinely collect ethnicity data which limits our interpretation on study inclusivity and generalisability. A future RCT should recruit from sites in geographical areas that serve diverse populations and work in collaboration with community groups to ensure the study is accessible and inclusive for all potential participants. Working with community groups, we have co-developed guidance on inclusive approaches to involvement of community groups in health research,^49^ and this should be followed to optimise accessibility and develop culturally appropriate study processes and documents. Another limitation is that we did not provide participants with an easy-read patient information leaflet (PIL) and/or information video; it is increasingly now acknowledged that standard PILs are lengthy, inappropriately complex and have poor readability, which can have a negative impact on people’s comprehension of the information provided.^50^ To mitigate this barrier to participation, a future RCT would need to work with diverse patients and communities to develop an accessible PIL to improve inclusivity.

To our knowledge, this is the first study to assess the feasibility of an RCT to evaluate the clinical and cost-effectiveness of pre-operative exercise and protein supplementation for frail patients undergoing primary THR or TKR in the NHS. Two previous feasibility studies from The Netherlands involving 3–6 weeks of pre-operative physiotherapy for frail patients reached differing conclusions on the feasibility of an RCT,^51 52^ however the intervention in both studies was exercise alone without protein supplementation. A protocol has been published for a feasibility study in Canada investigating a multicomponent prehabilitation intervention comprising exercise, protein supplementation, vitamin D supplementation and medication review for frail individuals undergoing joint replacement.^53^ Further studies evaluating prehabilitation interventions are needed to generate a robust evidence base to inform healthcare services.

By undertaking feasibility work to address key uncertainties, this study has generated important data on the likely success of a future RCT and provided insight into approaches to optimise trial design and processes. A future RCT would provide evidence to guide decisions by patients, clinicians and policymakers and inform service provision. If proven clinically and cost-effective, integrating a prehabilitation intervention into usual NHS care could optimise health before surgery and improve outcomes for frail patients undergoing joint replacement. This is a particularly pertinent issue in the current NHS climate. Waiting lists for joint replacement are long and hospitals have different strategies to try and reduce waiting lists (eg, through independent sector treatment service providers or short stay pathways) and these often discriminate against frail patients as fitter and healthy patients are chosen for quicker pathways. A prehabilitation intervention has the potential to increase health and function while on the waiting list and reduce discrimination by making more frail patients eligible for these quicker pathways to surgery. An RCT is now needed to evaluate the clinical and cost-effectiveness of an exercise and protein supplementation prehabilitation programme for frail patients undergoing joint replacement.

## supplementary material

10.1136/bmjopen-2024-084678 online supplemental file 1

## Data Availability

Data are available in a public, open access repository.
