# Supplementary material for: Prehabilitation for frail patients undergoing hip and knee replacement in the UK: Joint PREP feasibility study for a randomised controlled trial
Source: BMJ Open. 2024 Sep 17;14(9):e084678. doi: 10.1136/bmjopen-2024-084678 (PMC11409239; doi:10.1136/bmjopen-2024-084678)
Supplement: Supplementary file 2 [file bmjopen-2024-084678.reviewer_comments.pdf]

PEER REVIEW HISTORY

BMJ Open publishes all reviews undertaken for accepted manuscripts. Reviewers are asked to complete a checklist review form and are provided with free text boxes to elaborate on their assessment. These free text comments are reproduced below.

ARTICLE DETAILS

**TITLE (PROVISIONAL)**	Prehabilitation for frail patients undergoing hip and knee replacement in the UK: Joint PREP feasibility study for a randomised controlled trial
**AUTHORS**	Khalid, Tanzeela; Ben-Shlomo, Yoav; Bertram, Wendy; Culliford, Lucy; Henderson, Emily; Jepson, Marcus; Johnson, Emma; Mitchell, Alex; Palmer, Shea; Evans, Jonathan; Whitehouse, Michael; Wylde, Vikki

VERSION 1 - REVIEW

**REVIEWER NAME**	***Punnoose, Anuj***
**REVIEWER AFFILIATION**	Cambridge University Hospitals NHS Foundation Trust, Physiotherapy Department
**REVIEWER CONFLICT OF INTEREST**	na
**DATE REVIEW RETURNED**	06-Feb-2024

**GENERAL COMMENTS**	I appreciate the opportunity to review this manuscript, and I would like to express my gratitude for including me in the process. The subject matter, focusing on prehabilitation within Orthopaedics, is highly relevant and demands well-executed studies. I commend all the authors for their dedicated efforts in successfully completing this study.<br><br>I have a few points and suggestions as below;<br><br>Abstract<br>14 Which Three hospitals- NHS or Private. Hospital or Elective centre. Needs to be specific here<br><br>27 The authors need to expand the methods section here. Where are the outcome measures?. How did they capture the data? What qualitative work did they perform and how did they analyse the data?<br><br>37 Nineteen participants- What is the percentage here. If you are reporting numbers and percentages in results please do so consistently throughout.<br><br>43 Same as above. How many returned the questionnaire?<br><br>44 Same as above<br><br>52 Please report on Ethics and Dissemination<br><br><br>Background<br>6 Please change the sentence to '… chronic pain and disability

	globally'.<br><br>18-21 This sentence doesn't make any sense. You may want to change it to ' An ageing population alongside an increased surgical waiting list could have a likely increase in frailty among patients undergoing…'. Please also explain why this might be the case here e.g. chronicity, deconditioning and sarcopenia?<br><br>47-51 There is no reference to any previous studies on prehab in this population. What is the justification for this study.?<br><br>Methods<br><br>Page 7 line 29 Where is the baseline questionnaire? Please bring the questionnaire section from page 9 to below this paragraph and kindly rename the title to 'Outcomes' as Questionnaires could mean either screening or baseline/follow-up questionnaires<br><br>Page 7 line 36 How was allocation concealment ensured? It is important to explain this method here. e.g. All study personnel was blinded to the allocation sequence. When did you randomise the patient?. Is it before or after giving out baseline questionnaires. If participants are allocated after baseline evaluation, there's a risk that the group assignments could be influenced by the knowledge of individual participants' baseline characteristics, and this could introduce bias into the study.<br><br>Page 7 Intervention- The authors explain here that the patients had one session with the Physiotherapist and then followed a personalised programme with follow-up telephone calls. So how did the physiotherapist decide on the dosage, intensity etc and what was the criteria for progression or regression? Kindly elaborate<br><br>Page 8 Line 31 Did you provide patients with therabands of varying resistance or the same for everyone. How did the therapist decide on this?<br><br>Page 9 Questionnaires- How were the questionnaires send- Is it by Post or via REDcap?<br>Are you planning to capture some objective outcome measures like muscle strength or the Short Physical Performance Battery tests in your RCT? If yes, why did you not assess its feasibility at this stage?<br><br>Page 11 Analysis- Were the transcripts reviewed by participants? or how did you ensure credibility of data?<br><br>Page 14 line 56- So the followup was 12 weeks after randomisation- Is this pre-op? Do you have any or planning any post-op follow-ups in the future RCT? If not, why? as ultimately you want to assess if prehabilitation had any effect on post-operative outcomes too? If yes, what time points are you planning for?<br><br>Page 15 Discussion- Please clearly state the limitations of the study e.g. sample size, unblinded

**REVIEWER NAME**	***Working, Zachary***
**REVIEWER AFFILIATION**	Oregon Health and Science University Foundation
**REVIEWER CONFLICT OF INTEREST**	Na

**DATE REVIEW RETURNED**	01-Mar-2024

**GENERAL COMMENTS**	I was asked to review "Prehabilitation for frail patients undergoing hip and knee replacement: Joint PREP feasibility study for a randomised controlled trial" as a feasibility study for a rehabilitation & protein supplementation RCT after joint replacement.<br><br>When I review feasibility studies I review first to consider: did the authors do what they set out to do? I think this feasibility study is done well. They set out to identify challenges in executing a specific trial design of randomization & PRO dominant outcomes. Now they have numbers that will help them appropriately budget and scope their trial which is highly useful and their pilot is completed and that is a massive amount of work. I applaud their efforts.<br><br>I also read these with the thought: what do I think about the trial design? This matters. Research dollars are scarce. They can't all be done. I have complaints in that the outcomes proposed for this trial are exclusively PRO based, when the interventions don't really just match PROs. These are physical interventions and there are opportunities for physical outcomes. Patients should perhaps perform timed-up-and-go tests, or under the guise of rehabilitation perform tasks at the start and end of the trial period, and compare against testing for the non-rehabilitation group.<br><br>I think not including something other than surveys is a mistake and an efficiency miss. I would ask that the authors either explain why those types of outcomes aren't pursued or to discuss further opportunities in trial design.

**REVIEWER NAME**	***Smeets, Annemie***
**REVIEWER AFFILIATION**	KU Leuven
**REVIEWER CONFLICT OF INTEREST**	Na
**DATE REVIEW RETURNED**	12-Mar-2024

**GENERAL COMMENTS**	**General comments:**<br>I want to comment the authors with their extensive feasibility study. It is very complete and provides valuable insights in the barriers for pre-operative programs in frail patients.<br>I have some specific comments, mainly to further clarify uncertainties.<br><br>**Abstract**<br>Can the authors explain which treatment the control group received?<br><br>**Background**<br>Line 14-16: the authors state that an important part of the patients undergoing THR or TKR have frailty. Can they provide a bit more information on this term to ensure that readers know what they mean with this. For example the definition, and how this is evaluated/classified (low-moderate -severe)?<br>Line 52-53: do the authors had any key uncertainties in mind? Why do they think that such RCT might not be feasible? Concrete examples will justify this feasibility study.<br><br>**Methods**

	Page 7 – Line 17: can you describe how patient identification was performed? Eg. was this done by screening medical records? Or at the consultation of a specific department?<br>Were all questionnaires sent by post? Or digitally?<br>Page 7 – Line 48: did the investigators asked which aspects of usual care were received by the patient?<br>Page 8 – line 10: did the patients had only 1 appointment with a physiotherapist? Or were there follow-up appointments as well?<br>Page 9 – Line 3: can the authors provide more information of the physiotherapists that were contacted to participate? How many physiotherapists delivered the training?<br>Page 10 – Line 29: Patients had to keep a log book to record their daily activities and protein intake. Do you have any evidence that this is a valid and reliable way in elderly? Do you have any measures on how well this was done?<br>Please also include this as an important limitation of the study.<br>Page 10 – Line 48-50: Can the authors further explain this sentence? What do they mean with this?<br><br>**Results**<br>Page 11 - Line 58: why were not all 79 patients who provided informed consent randomised?<br>Page 12 - Line 52: only 19 patients completed the adherence logs. Therefore, I would not use the word 'Overall' as you don't know what the 7 patients who did not fill in the logs did. Suggest to write 'of these 19 patients, 10 adhered..<br><br>**Discussion**<br>The authors conclude that the adherence rate of 53% is acceptable/good to proceed to the full RCT. For me, this adherence rate seems quite low. On what did the authors based to decide that this is an acceptable adherence rate?<br>The authors focused on whether the patients did like the protein food/drink. Do they also have insights on the beliefs of the patients regarding the effectiveness of this supplement? If they are well informed on the benefits, they might see the benefits above the bad taste?

**REVIEWER NAME**	***Toyooka, Takeshi***
**REVIEWER AFFILIATION**	Nishikawa Orthopaedics Clinic, rehabilitation
**REVIEWER CONFLICT OF INTEREST**	Na
**DATE REVIEW RETURNED**	26-Mar-2024

**GENERAL COMMENTS**	The qualitative research based on the interviews was considered a very advanced and valuable study.<br>On the other hand, it is questionable whether the following modifications could not be applied to future RCTs from this interview.<br>Participants were expected to have cognitive concerns, as they made statements that seemed to indicate that they were unable to determine which group they belonged to.<br>Considering that the participants were elderly, it was possible that whether they were able to accurately carry out the self-report without forgetting to take their supplements could affect the current study.<br>It was also considered desirable to have the usual care group also take a dummy supplement to avoid judgment of which group they

	belonged to based on communication between participants.<br>On the other hand, since the recruitment, Adherence and retention rates met the targets, the quantitative results were not considered problematic for the conduct of the study.

**REVIEWER NAME**	***Gould, Daniel***
**REVIEWER AFFILIATION**	The University of Melbourne, Department of Surgery
**REVIEWER CONFLICT OF INTEREST**	Na
**DATE REVIEW RETURNED**	17-Apr-2024

**GENERAL COMMENTS**	Thank you for the opportunity to review this manuscript. It is obvious that this comprises a critical step in an important and extensive body of work, but standing on its own two feet it is an exceptional paper that I believe will be of interest to readers of this journal as well as both patients and practitioners experiencing total joint replacement. I believe the authors have clearly demonstrated the feasibility of their planned RCT, and I commend them on their efforts to prioritise patient involvement throughout the design and implementation process.<br>I have some comments for consideration to improve the clarity of the manuscript prior to publication. I recognise that some of these points are likely expanded upon, in detail, in the RCT protocol, and as such I will leave it to the authors' discretion as to whether they feel it is appropriate to expand upon certain points in this feasibility study manuscript in order to make it more self-contained, or provide reference to the protocol.<br>- Opening paragraph of 'Background' section: suggets providing the working definition for 'frailty' used in this study, after the sentence on average age of people having joint replacement in the UK. This seems like a fitting place to provide a brief definition of this key terminology to help orientate readers.<br>- Suggest providing a brief description of the GFI when it is first mentioned.<br>- Suggest providing a brief description of the NEMEX-TJR programme when it is first mentioned<br>- The panel of questionnaires presented to patients seems burdensome. Perhaps it would be worth briefly reiterating the rationale for including several different questionnaires - i.e. the information the authors were aiming to obtain from these questionnaires and why it was deemed necessary to include all of them. I feel this is a valuable addition to this manuscript reporting the findings of the feasibility study, in which it is especially pertinent to consider the pragmatic aspects including burden on participants.<br>- Suggest expanding upon rationale, and/or providing reference, for the criteria by which a patient was judged to have adhered to the intervention in the Methods subsection 'Feasibility outcomes'.<br>- Suggest expanding on the 'Sample size' subsection of the Methods. It is not clear to me why these numbers (200 eligible patients, etc.) were selected.

**REVIEWER NAME**	***Kabali, Conrad***
**REVIEWER AFFILIATION**	Health Quality Ontario
**REVIEWER CONFLICT OF INTEREST**	Na
**DATE REVIEW RETURNED**	30-Apr-2024

**GENERAL COMMENTS**	Page 10, Sample Size: Kindly elaborate the source of the ±6.35% confidence interval value. Additionally, please provide the rationale underlying the choice of a 30% recruitment rate.<br>Page 11, Progression Criteria: Kindly provide rationale for the selection of the thresholds for the "progression criteria."

**REVIEWER NAME**	***Gumedze, Freedom***
**REVIEWER AFFILIATION**	University of Cape Town, Statistical Sciences
**REVIEWER CONFLICT OF INTEREST**	Na
**DATE REVIEW RETURNED**	17-May-2024

**GENERAL COMMENTS**	The manuscript presents the results of a feasibility study.The objective of the feasibility study was to determine the feasibility of conduction a RCT to evaluate a prehabilitation programme for frail patients undergoing total hip replacement (THR) or total knee replacement (TKR).<br><br>The study recruited and randomised 64 patients from 3 hospitals. The study participants were adults aged at least 65 years years old who have to be frail and were scheduled for pimary THR or TKR. The study outcomes were Eligibility and recruitment rates, intervention adherence, data completion rates, retention rates and acceptability of the trial and intervention. Based on the results of their study, the authors demonstrate that a RCT on this topic is feasible.<br><br>The feasibility study was a well designed and executed feasibility study following the appropriate protocols for design, conduction and reporting the results of a feasibility study.<br>The manuscript is well written and the results are well presented. I recommend a acceptance of the manuscript after a minor revision. The authors must address the following comments:<br><br>1. Pages 11-15: The reported study outcomes must be presented with confidence intervals.<br><br>2. Table 2 and Discussion: The authors have acknowledged that the study sites served populations with limited ethnic diversity so a future RCT should recruit participants from study areas with diverse populations. The acknowledgement of this short coming is commended.

VERSION 1 – AUTHOR RESPONSE

**Reviewer 1 comments**	**Authors' response**
I appreciate the opportunity to review this manuscript, and I would like to express my gratitude for including me in the process. The	Thank you for your positive and supportive feedback.

subject matter, focusing on prehabilitation within Orthopaedics, is highly relevant and demands well-executed studies. I commend all the authors for their dedicated efforts in successfully completing this study.	
Abstract	
14 Which Three hospitals- NHS or Private. Hospital or Elective centre. Needs to be specific here	We have clarified that the study was conducted in three NHS hospitals (page 2).
27 The authors need to expand the methods section here. Where are the outcome measures?. How did they capture the data? What qualitative work did they perform and how did they analyse the data?	We have added further details to the abstract to clarify that the outcome measures were patient-reported outcome measures and that qualitative interviews were conducted with participants and non-participants and analysed using thematic analysis (page 2).
37 Nineteen participants- What is the percentage here. If you are reporting numbers and percentages in results please do so consistently throughout.	Thank you, we have now added this percentage to the results section of the abstract (page 2).
43 Same as above. How many returned the questionnaire?	We have now added this percentage (page 2).
44 Same as above	Thank you for the suggestion, however the interview sample comprised patients who did and did not participate in the feasibility study, and therefore providing a percentage here would be less useful to readers.
52 Please report on Ethics and Dissemination	We have added an ethics approval statement to the abstract (page 3). We have not added a dissemination statement as dissemination is through this article.
Background	
6 Please change the sentence to '… chronic pain and disability globally'.	We have now made this change (page 4).
18-21 This sentence doesn't make any sense. You may want to change it to ' An ageing population alongside an increased surgical waiting list could have a likely increase in frailty among patients undergoing…'. Please also explain why this might be the case here e.g. chronicity, deconditioning and sarcopenia?	Thank you for highlighting this, we have revised the sentence to "A future increase in the prevalence and severity of frailty among patients having joint replacement in the NHS is likely due to an ageing population and the deterioration in health associated with a long surgical waiting time" (page 4).
47-51 There is no reference to any previous studies on prehab in this population. What is the justification for this study.?	We have now expanded on paragraph 2 in the introduction to discuss the existing literature on prehabilitation for frail patients and the proof of concept to justify this study. This includes studies that have demonstrated the effectiveness of prehabilitation for frail patients in a surgical content. We have also clarified that prehabilitation for patients having hip and knee replacement has not yet been evaluated (pages 4-5).
Methods	

Page 7 line 29 Where is the baseline questionnaire? Please bring the questionnaire section from page 9 to below this paragraph and kindly rename the title to ‘Outcomes’ as Questionnaires could mean either screening or baseline/follow-up questionnaires	Thank you, we agree this requires clarification. To maintain the flow of the methods section, we have removed reference to the baseline questionnaire from the recruitment section (page 7) and have clarified in the questionnaires section that the questionnaires were completed at baseline (before randomisation) and 12 weeks after randomisation (page 9).
Page 7 line 36 How was allocation concealment ensured? It is important to explain this method here. e.g. All study personnel was blinded to the allocation sequence. When did you randomise the patient?. Is it before or after giving out baseline questionnaires. If participants are allocated after baseline evaluation, there's a risk that the group assignments could be influenced by the knowledge of individual participants' baseline characteristics, and this could introduce bias into the study.	By using REDCap, the allocation sequence was concealed from all study personnel until potential participant’s eligibility had been established, including staff recruiting patients into the study, therefore ensuring that the allocation could not be predicted prior to randomisation and introduce bias into the study. We have clarified this in the manuscript (page 7).<br><br>We have clarified on page 9 that baseline questionnaires were completed before randomisation. There was no risk that knowledge of patients baseline characteristics would influence the outcome of randomisation as the allocation sequence was concealed from all study personnel.
Page 7 Intervention- The authors explain here that the patients had one session with the Physiotherapist and then followed a personalised programme with follow-up telephone calls. So how did the physiotherapist decide on the dosage, intensity etc and what was the criteria for progression or regression? Kindly elaborate	Thank you for this suggestion. In the initial appointment, the physiotherapist discussed each exercise with the participant and the participant was encouraged to decide which level of each exercise was most appropriate for them. They then performed them with the physiotherapist who checked safety and technique. Participants were encouraged to aim for 10-15 repetitions of each exercise and 2-3 sets. If they reported in a telephone follow-up call that they could confidently perform the exercises at one level, they were encouraged to consider progression to the next level. If participants reported that they were struggling with any of the exercises they were encouraged to regress to an easier level. We have elaborated on this in the manuscript (page 8).
Page 8 Line 31 Did you provide patients with therabands of varying resistance or the same for everyone. How did the therapist decide on this?	Participants were issued with one of four resistance bands (special heavy, extra heavy, heavy or medium) based on their individual capability. We have added this to the manuscript on page 8.
Page 9 Questionnaires- How were the questionnaires send- Is it by Post or via REDcap?<br>Are you planning to capture some objective outcome measures like muscle strength or the Short Physical Performance Battery tests	All questionnaires were administered by post, we have added this to page 9.<br><br>Our choice of patient-reported outcome measures to assess function reflects that the lack of patient-centred outcomes is a limitation

in your RCT? If yes, why did you not assess its feasibility at this stage?	of previous trials evaluating interventions for frailty. While we acknowledge that more objective assessments of function are valuable outcomes, our PPI group recommended that self-report function would be the most meaningful outcome to patients in a future RCT. We have discussed this as a study limitation on page 17.
Page 11 Analysis- Were the transcripts reviewed by participants? or how did you ensure credibility of data?	The transcripts were not reviewed by participants but were checked against original recordings for accuracy. To further ensure the credibility of the data we engaged in several strategies.<br><br>Firstly, two authors (EJ & MJ), both who have extensive qualitative research experience, met regularly to discuss, review, revise and agree the development of key themes. Secondly, to further ensure credibility and enhance the quality of our analysis and interpretations we paid close attention to negative or contradictory cases. Thirdly, to enrich understandings, we also discussed the key qualitative themes with our patient group. A summary of this process has been added to the manuscript on page 12.
Results	
Page 14 line 56- So the followup was 12 weeks after randomisation- Is this pre-op? Do you have any or planning any post-op follow-ups in the future RCT? If not, why? as ultimately you want to assess if prehabilitation had any effect on post-operative outcomes too? If yes, what time points are you planning for?	The 12 week follow-up was a pre-operative assessment timepoint. Our aim was to assess the feasibility of the study and the acceptability of the intervention. We have demonstrated in our previous orthopaedic trials that we can collect outcome data from patients after surgery and therefore this was not an aim of this feasibility study. We have expanded our introduction to discuss the existing literature which provides proof of concept that prehabilitation can improve outcomes after surgery (page 4). In the future RCT, we plan to assess outcomes every 3 months up to 12 months after randomisation, which will likely capture at least post-operative assessment for most participants in the trial.
Discussion	
Page 15 Discussion- Please clearly state the limitations of the study e.g. sample size, unblinded	We have included an additional paragraph in the discussion to more fully discuss the limitations of the study, including the sample size, lack of blinding, the use of patient-reported outcome measures, not collecting data on the usual pre-operative care that participants received, and

	assessment of intervention adherence (pages 16-17).

**Reviewer 2 comments**	**Authors' response**
When I review feasibility studies I review first to consider: did the authors do what they set out to do? I think this feasibility study is done well. They set out to identify challenges in executing a specific trial design of randomization & PRO dominant outcomes. Now they have numbers that will help them appropriately budget and scope their trial which is highly useful and their pilot is completed and that is a massive amount of work. I applaud their efforts.<br><br>I also read these with the thought: what do I think about the trial design? This matters. Research dollars are scarce. They can't all be done. I have complaints in that the outcomes proposed for this trial are exclusively PRO based, when the interventions don't really just match PROs. These are physical interventions ad there are opportunities for physical outcomes. Patients should perhaps perform timed-up-and-go tests, or under the guise of rehabilitation perform tasks at the start and end of the trial period, and compare against testing for the non-rehabilitation group.<br><br>I think not including something other than surveys is a mistake and an efficiency miss. I would ask that the authors either explain why those types of outcomes aren't pursued or to discuss further opportunities in trial design.	Thank you for the positive and supportive comments on our feasibility study.<br><br>In relation to the focus on patient-reported outcomes, this was based on feedback from our PPI group that the outcomes that matter the most to patients are mobility and physical ability to do activities of daily living and the most meaningful way to assess this outcome is using a patient-reported outcome measure. A limitation of previous trials evaluating interventions for frailty is a lack of person-centred outcomes; we aimed to address this by being guided by patients in our selection of outcomes. However, we appreciate the value that objective measures can add, and in our main trial (for which we are currently preparing a grant application) we plan to include a measure of muscle strength to evaluate the mechanism of action of the intervention. We have discussed our choice of outcomes in the limitations section of our discussion (pages 16-17).

**Reviewer 3 comments**	**Authors' response**
I want to comment the authors with their extensive feasibility study. It is very complete and provides valuable insights in the barriers for pre-operative programs in frail patients.<br>I have some specific comments, mainly to further clarify uncertainties.	Thank you for your supportive and positive comments.
Abstract	
Can the authors explain which treatment the control group received?	We did not collect detailed information on usual care, and we have acknowledged this as a study

	limitation in the discussion (pages 16-17). On page 7 we provide a broad overview of usual care, explaining that this comprises clinical review by a surgeon before and after surgery but that other aspects of usual care varied between NHS Trusts, but could involve education classes, physiotherapy, and occupational therapy.
Background	
Line 14-16: the authors state that an important part of the patients undergoing THR or TKR have frailty. Can they provide a bit more information on this term to ensure that readers know what they mean with this. For example the definition, and how this is evaluated/classified (low-moderate -severe)?	Thank you for this helpful suggestion, we have now added a sentence to the background to explain that frailty is an age-related condition associated with a deterioration in the physiological capacity of multiple organ systems that causes an increased susceptibility to stressors, such as illness and surgery (page 4).
Line 52-53: do the authors had any key uncertainties in mind? Why do they think that such RCT might not be feasible? Concrete examples will justify this feasibility study.	Previous RCTs of interventions with an exercise component have highlighted that intervention adherence can be an issue and this was one of the key uncertainties that we wanted to address in this study. We have clarified this in the background on page 5.
Methods	
Page 7 – Line 17: can you describe how patient identification was performed? Eg. was this done by screening medical records? Or at the consultation of a specific department? Were all questionnaires sent by post? Or digitally?	Thank you, we have now clarified that hospital records were searched to identify patients who were aged 65 or over and had more than 12 weeks until the intended date for their TKR or THR operation. The second stage of screening for frailty was completed using the self-completed GFI, which was sent to patients by post (page 6).
Page 7 – Line 48: did the investigators asked which aspects of usual care were received by the patient?	We did not collect this information and we have acknowledged this as a study limitation in the discussion (pages 16-17).
Page 8 – line 10: did the patients had only 1 appointment with a physiotherapist? Or were there follow-up appointments as well?	Patients had one appointment with the physiotherapy and then follow-up was via telephone calls at 1, 2, 4, 6, 8 and 10 weeks. We have amended the manuscript to clarify that patients had “a single 1:1 appointment” on page 7.
Page 9 – Line 3: can the authors provide more information of the physiotherapists that were contacted to participate? How many physiotherapists delivered the training?	On page 9 we have added that the intervention was delivered by physiotherapists who worked clinically with orthopaedic patients and that 2 physiotherapists at each hospital site were trained to deliver the intervention.
Page 10 – Line 29: Patients had to keep a log book to record their daily activities and protein intake. Do you have any evidence that this is a valid and reliable way in elderly? Do you have any measures on how well this was done? Please also include this as an important limitation of the study.	Thank you for raising this important limitation. There is not a consistent tool to measure adherence to exercise interventions, and diaries and logs are the most frequently used tools. We acknowledge that collecting robust data on adherence to home-based interventions is challenging as self-reported adherence data is open to recall bias and overestimation of

	adherence. We have reflected on this as a limitation of our study. We have also acknowledged that our assessment of intervention adherence was limited as not all participants in the intervention group returned their self-completed adherence log (pages 16-17).
Page 10 – Line 48-50: Can the authors further explain this sentence? What do they mean with this?	Apologies for the confusion. To meet our target of 60 participants (deemed appropriate for feasibility studies) with an estimated recruitment rate of 30%, we estimated we would need to identify 200 eligible people. If we assumed 40% of those screened would be pre-frail/frail and potentially eligible, we expect to actively screen 500 patients for frailty. As this is a feasibility study, we based our sample size on recruitment rate. If we identified 200 eligible subjects, we could estimate a recruitment rate of 30% (i.e., 60 participants) to within a 95% confidence interval (CI) of ±6.35%. We have now clarified this on page 11.
Results	
Page 11 - Line 58: why were not all 79 patients who provided informed consent randomised?	We provide the reasons that 15 patients who gave informed consent were not randomised are provided in the CONSORT diagram (figure 1). Ten patients withdrew their consent after prior to randomisation due to change of mind/declined/no response and five patients were not randomised as we had reached our recruitment target.
Page 12 - Line 52: only 19 patients completed the adherence logs. Therefore, I would not use the word 'Overall' as you don't know what the 7 patients who did not fill in the logs did. Suggest to write 'of these 19 patients, 10 adhered.	Thank you, we have amended to your suggested wording on page 13.
Discussion	
The authors conclude that the adherence rate of 53% is acceptable/good to proceed to the full RCT. For me, this adherence rate seems quite low. On what did the authors based to decide that this is an acceptable adherence rate?	Our adherence rate requires interpretation in the context of the study population, who were frail, older patients with chronic pain and functional limitations due to severe osteoarthritis. Therefore, we expected participants to experience barriers to participating in a multimodal and intensive prehabilitation intervention. We have now reflected on the need to consider methods to optimise adherence rates in a future RCT in the limitations section of the discussion (pages 16-17).
The authors focused on whether the patients did like the protein food/drink. Do they also have insights on the beliefs of the patients regarding the effectiveness of this	Thank you for your comment. We have revisited the data and can share that a few participants did reflect that supplementing their diet with additional protein could have potential benefits

supplement? If they are well informed on the benefits, they might see the benefits above the bad taste?	to their health and wellbeing. One participant also credited the changes that they had observed (improved mood and feeling better) to taking the supplements. However, we have insufficient insight to draw any clear or strong conclusions about the relationship between participant beliefs and their willingness to take the supplement, even if seen as undesirable due to the taste.

**Reviewer 4 comments**	**Authors' response**
The qualitative research based on the interviews was considered a very advanced and valuable study.<br>On the other hand, it is questionable whether the following modifications could not be applied to future RCTs from this interview.<br>Participants were expected to have cognitive concerns, as they made statements that seemed to indicate that they were unable to determine which group they belonged to.<br>Considering that the participants were elderly, it was possible that whether they were able to accurately carry out the self-report without forgetting to take their supplements could affect the current study.<br>It was also considered desirable to have the usual care group also take a dummy supplement to avoid judgment of which group they belonged to based on communication between participants.<br>On the other hand, since the recruitment, Adherence and retention rates met the targets, the quantitative results were not considered problematic for the conduct of the study.	Thank you for your positive comments and feedback on the study. To address the findings from the qualitative interviews relating to participant understanding of the study, we acknowledge that a limitation of the study was that we did not provide participants with an easy-read patient information leaflet and/or information video, and that this would be necessary for a future RCT to ensure that information about the trial is provided in accessible formats (page 17). We have also acknowledged the limitations of the self-report method of assessing adherence to the intervention (pages 16-17). We did not provide participants with a placebo supplement as it would not be possible to blind participants to treatment allocation because of the exercise component of the intervention. We have acknowledged that the lack of blinding of the intervention could potentially led to an overestimate of the treatment effect in a future RCT (pages 16-17).

**Reviewer 5 comments**	**Authors' response**
Thank you for the opportunity to review this manuscript. It is obvious that this comprises a critical step in an important and extensive body of work, but standing on its own two feet it is an exceptional paper that I believe will be of interest to readers of this journal as well as both patients and practitioners experiencing total joint replacement. I believe the authors have clearly demonstrated the feasibility of their planned RCT, and I commend them on their efforts to prioritise patient involvement throughout the design and implementation	Thank you for your very positive and support comments on this manuscript.

process. I have some comments for consideration to improve the clarity of the manuscript prior to publication. I recognise that some of these points are likely expanded upon, in detail, in the RCT protocol, and as such I will leave it to the authors' discretion as to whether they feel it is appropriate to expand upon certain points in this feasibility study manuscript in order to make it more self-contained, or provide reference to the protocol.	
Opening paragraph of 'Background' section: suggets providing the working definition for 'frailty' used in this study, after the sentence on average age of people having joint replacement in the UK. This seems like a fitting place to provide a brief definition of this key terminology to help orientate readers.	Thank you, we have now added a definition of frailty to the background (page 4).
Suggest providing a brief description of the GFI when it is first mentioned.	We have now added additional details about the GFI to explain that it is a 15-item multidimensional screening tool for frailty, with questions covering daily activities, health problems and psychosocial functioning (page 6).
Suggest providing a brief description of the NEMEX-TJR programme when it is first mentioned	We have added that the NEMEX-TJR programme is a neuromuscular training method for patients with osteoarthritis who are undergoing joint replacement, and added a reference to a relevant study of the NEMEX-TJR programme in patients listed for joint replacement on page 8.
The panel of questionnaires presented to patients seems burdensome. Perhaps it would be worth briefly reiterating the rationale for including several different questionnaires - i.e. the information the authors were aiming to obtain from these questionnaires and why it was deemed necessary to include all of them. I feel this is a valuable addition to this manuscript reporting the findings of the feasibility study, in which it is especially pertinent to consider the pragmatic aspects including burden on participants.	Thank for your raising this. The questionnaires were chosen to assess the range of health domains that could potentially be influenced by the intervention, including joint pain and function, general health, capability, frailty, physical activity levels, exercise self-efficacy and beliefs, dietary protein adequacy and BMI. We have added our justification for the selection of the questionnaires, and clarified the domains that were measured by each questionnaire on page 9.
Suggest expanding upon rationale, and/or providing reference, for the criteria by which a patient was judged to have adhered to the intervention in the Methods subsection 'Feasibility outcomes'.	Defining adherence is challenging and a recent systematic review identified no single definition of adherence to therapeutic exercises of musculoskeletal pain (https://pubmed.ncbi.nlm.nih.gov/29875278/). The rationale for our adherence criteria was to reflect current clinical opinion that multimodal exercise, performed at least 3 times per week for at least 2 weeks before surgery appears to show the most promise for

	improving post-surgical outcomes in frail patients (https://pubmed.ncbi.nlm.nih.gov/32384334/). We now added this to the feasibility outcomes section of the methods (page 11).
Suggest expanding on the 'Sample size' subsection of the Methods. It is not clear to me why these numbers (200 eligible patients, etc.) were selected.	Apologies for the confusion. To meet our target of 60 participants (deemed appropriate for feasibility studies) with an estimated recruitment rate of 30%, we estimated we would need to identify 200 eligible people. If we assumed 40% of those screened would be pre-frail/frail and potentially eligible, we expect to actively screen 500 patients for frailty. As this is a feasibility study, we based our sample size on recruitment rate. If we identified 200 eligible subjects, we could estimate a recruitment rate of 30% (i.e., 60 participants) to within a 95% confidence interval (CI) of ±6.35%. We have now clarified this on page 11.

**Reviewer 6 comments**	**Authors' response**
Page 10, Sample Size: Kindly elaborate the source of the ±6.35% confidence interval value. Additionally, please provide the rationale underlying the choice of a 30% recruitment rate.	Apologies for the confusion. To meet our target of 60 participants (deemed appropriate for feasibility studies) with an estimated recruitment rate of 30%, we estimated we would need to identify 200 eligible people. If we assumed 40% of those screened would be pre-frail/frail and potentially eligible, we expect to actively screen 500 patients for frailty. As this is a feasibility study, we based our sample size on recruitment rate. If we identified 200 eligible subjects, we could estimate a recruitment rate of 30% (i.e., 60 participants) to within a 95% confidence interval (CI) of ±6.35%. We have now clarified this on page 11. We have also added our justification for our chosen progression criteria on page 11.
Page 11, Progression Criteria: Kindly provide rationale for the selection of the thresholds for the "progression criteria."	Thank you, we have expanded on the rationale for our progression criteria. It has recently been recommended that the rationale for progression criteria need not be statistical as feasibility studies are usually underpowered for hypothesis testing, but that the rationale can be based on feasibility objectives, clinical or contextual assumptions, pragmatically derived, based on previous feasibility or observational work, developed using consensus methods involving a range

	of stakeholders (https://pubmed.ncbi.nlm.nih.gov/37061720/). We have now clarified that our progression criteria were pragmatically derived by the research team to demonstrate that recruitment, adherence and retention would be sufficient to deliver a future RCT (pages 11-12).

**Reviewer 7 comments**	**Authors' response**
The feasibility study was a well designed and executed feasibility study following the appropriate protocols for design, conduction and reporting the results of a feasibility study. The manuscript is well written and the results are well presented. I recommend a acceptance of the manuscript after a minor revision. The authors must address the following comments:	Thank you for your positive comments and encouragement.
Pages 11-15: The reported study outcomes must be presented with confidence intervals.	Thank you, we have now amended this and report 95% confidence intervals alongside the means in the tables.
Table 2 and Discussion: The authors have acknowledged that the study sites served populations with limited ethnic diversity so a future RCT should recruit participants from study areas with diverse populations. The acknowledgement of this short coming is commended.	Thank you, we agree that this is very important for the main trial (and clinical research more broadly) and alongside selecting sites that serve diverse populations, we will also prioritise work to improve the inclusivity and accessibility of the trial.

Additional changes

We have added Jonathan Evans as a co-author, who was inadvertently not included as a co-author in the original submission. We have explained how Jonathan meets the authorship criteria in the 'author contribution' section.

VERSION 2 – REVIEW

**REVIEWER NAME**	***Punnoose, Anuj***
**REVIEWER AFFILIATION**	Cambridge University Hospitals NHS Foundation Trust, Physiotherapy Department
**REVIEWER CONFLICT OF INTEREST**	Na
**DATE REVIEW RETURNED**	07-Aug-2024

**GENERAL COMMENTS**	Many thanks for asking me to re-review the manuscript. The authors seem to have addressed all the major recommendations.

**REVIEWER NAME**	***Smeets, Annemie***
**REVIEWER AFFILIATION**	KU Leuven
**REVIEWER CONFLICT OF INTEREST**	Na
**DATE REVIEW RETURNED**	05-Aug-2024

**GENERAL COMMENTS**	The authors made a good job in addressing all comments. The manuscript was greatly improved and all my comments were answered in detail.<br>I have no additional concerns, but I would recommend the authors to also capture what patients do as 'usual care'.

**REVIEWER NAME**	***Gould, Daniel***
**REVIEWER AFFILIATION**	The University of Melbourne, Department of Surgery
**REVIEWER CONFLICT OF INTEREST**	Na
**DATE REVIEW RETURNED**	26-Jul-2024

**GENERAL COMMENTS**	I commend the authors on their detailed and thoughtful responses my comments as well as the corresponding changes made to the manuscript. I also read through their responses to the Editors and the other reviewers, and I similarly commend the authors on what I imagine was an extensive revision process. I am satisfied that my comments have been addressed and I have nothing further to add.
